# Idiopathic Intrahepatic Cholestasis as an Unusual Presentation of Hodgkin's Disease

**DOI:** 10.1155/2015/987860

**Published:** 2015-06-18

**Authors:** Hande Atalay, Banu Boyuk, Muhammet Ates, Aslan Celebi, Ismail Ekizoglu, Arzu A. Didik, Aysenur A. Igdem

**Affiliations:** ^1^Department of Internal Medicine, Gaziosmanpasa Taksim Education and Research Hospital, 34240 Istanbul, Turkey; ^2^Department of Pathology, Gaziosmanpasa Taksim Education and Research Hospital, 34240 Istanbul, Turkey

## Abstract

Intrahepatic cholestasis in the form of paraneoplastic phenomena is an uncommon presentation of Hodgkin's lymphoma (HL). Herein we report the diagnosis of mixed type HL-related idiopathic intrahepatic cholestasis in a 73-year-old man presenting with jaundice, after the inguinal lymph node biopsy indicative of mixed cellular type HL and liver biopsy consistent with intrahepatic cholestasis, following several diagnostic interventions including surgery for suspected extrahepatic obstructive cholestasis. Our findings emphasize the value of early liver biopsy in the diagnostic algorithm along with consideration of HL-related idiopathic intrahepatic cholestasis as a diagnosis of exclusion in cholestatic jaundice of obscure origin.

## 1. Introduction

The incidence of hepatic involvement at the time of diagnosis of Hodgkin's lymphoma (HL) was reported to range from 3% to 14%, along with much higher range of 38%–66% of incidence documented in postmortem series [1–4]. Although jaundice is not an uncommon symptom in patients with Hodgkin's disease identified in 3% to 68% of patients, based on previous studies, it can be concluded that jaundice is likely to develop during follow-up in most of the cases [3, 5], whereas cholestasis as the presenting symptom of HL is reported to be uncommon (<4%) [3].

Direct intrahepatic parenchymal or biliary epithelial involvement of HL has been considered to be the most common reason for hepatic dysfunction in patients with HL [5], while extrahepatic bile duct obstruction secondary to lymphadenopathy as well as certain comorbid conditions unrelated to liver involvement by HL such as hemolytic anemia, infectious hepatitis, previous underlying liver disease, or drug toxicities has also been documented in the pathogenesis of HL-related cholestasis [1, 3, 5, 6]. In the years following first described by Bouroncle in 1962, development of intrahepatic cholestasis as a paraneoplastic phenomenon has been reported in a small minority of patients with HL but without lymphomatous involvement of the liver and precipitating causative factors for liver dysfunction and classified into vanishing bile duct syndrome (VBDS) or idiopathic intrahepatic cholestasis based on the presence or absence of ductopenia, respectively [1, 3, 5–7].

To date, most cases of HL-related idiopathic cholestasis have been associated with VDBS in the literature, while HL-related idiopathic cholestatic hepatitis without a paucity of small intrahepatic bile ducts has been considered as a rare phenomenon being described only in a few reports [5, 7]. A clear pathogenetic relationship between HL and idiopathic cholestasis cannot be identified. However the concomitance of the two diseases makes the hypothesis of idiopathic intrahepatic cholestasis likely to be a paraneoplastic manifestation of HL. Clinical outcome is hepatic failure unless the underlying disease can be appropriately cured. Herein, we report a patient who presented with jaundice and subsequently diagnosed with HL who had clinical, laboratory, and pathological findings consistent with idiopathic intrahepatic cholestasis as an unusual presentation of Hodgkin's disease.

## 2. Case Report

**Figure 1:**
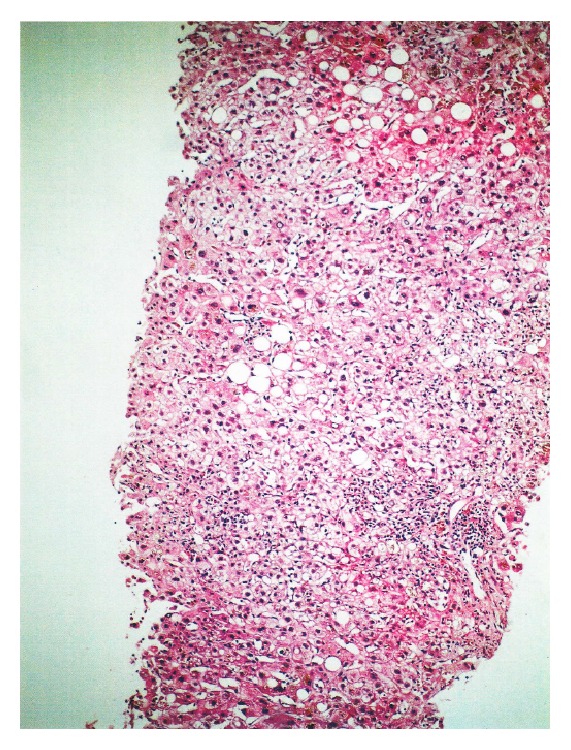
A portal tract showing prominent infiltration of mononuclear cells with sparse polymorphic leucocytes. Existence of extensive bile pigments in parenchyma and mild steatosis in portal tracts is striking (hematoxylin-eosin stain; original magnification ×10).

A 73-year-old man was admitted to our department with a 4-month history of pruritus and 2-month history of jaundice, alcoholic stools, dark urine associated with malaise, fatigue, and 8 kg weight loss. He had been admitted to the hospital for the complaint of pruritus in January 2012, while he was on routine follow-up for hypothyroidism but otherwise healthy. In March 2012, he had been hospitalized in general surgery department for further investigation upon findings of elevated liver enzymes and hyperbilirubinemia on laboratory tests (Table 1). No pancytopenia or bicytopenia was observed during the clinical course and ferritin levels were mildly elevated (not listed). Briefly, based on magnetic resonance cholangiogram findings suggestive of Klatskin tumor (Figure 1), following first percutaneous transhepatic cholangiography for drainage, he underwent cholecystectomy + T tube drainage + choleduct exploration, while no evidence of tumor was detected. Given that laboratory findings consistent with cholestasis persisted after surgery, an endoscopic retrograde cholangiopancreatography (ERCP) was performed, while revealing no bile duct obstruction or extrahepatic causes of cholestasis. Therefore, one month later the patient was referred to our department of internal medicine without any established diagnosis.

On admission to our department, physical examination findings revealed body temperature of 38.2°C along with 1 × 2 cm left inguinal lymphadenopathy and hepatomegaly. Liver size by percussion was determined to be 10 cm with no evidence of ascites. The spleen was not palpable and the abdomen was not tender with normal bowel sounds and an operational scar on the right abdominal quadrant. Cardiac and chest examination revealed normal findings.

**Figure 2:**
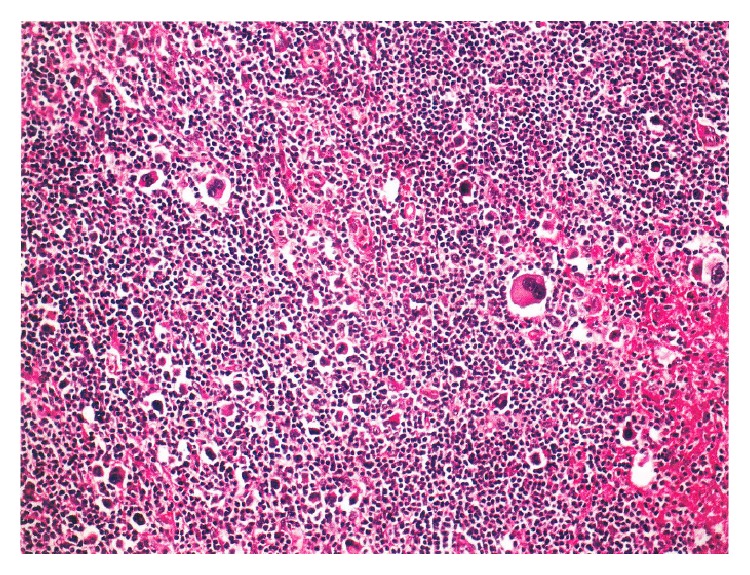
Inguinal lymph node examination shows mixed cellular type HD with Reed-Sternberg cells (hematoxylin-eosin stain; original magnification ×10).

There was no history of hepatitis, hepatotoxin exposure, intravenous (IV) drug abuse, or blood transfusion. Other than receiving thyroid hormone therapy for the last 10 years and oral secondary bile acid replacement for 2 months, he was free of any other medication or herbal remedies. As shown in Table 1, laboratory findings were consistent with cholestasis and hepatocellular injury which directed us to focus on intrahepatic causes of cholestasis. Serologic tests for hepatitis A, hepatitis B, and hepatitis C, cytomegalovirus, and HIV were all negative and antinuclear antibodies, antimitochondrial antibodies, anti-smooth muscle antibodies, and anti-liver-kidney microsomal antibody (anti LKM-1) were negative. Anti-ds DNA were also negative which is very rarely positive in autoimmune hepatitis. Abdominal ultrasound findings were normal except for hepatomegaly and operational findings. An abdominal computed tomography (CT) scan demonstrated hepatomegaly and multiple enlarged lymph nodes extending from the level of left paraaortic area to the left external iliac artery, while no lymphadenopathy was evident on thorax CT scan. The patient subsequently underwent ultrasound guided inguinal lymph node biopsy as well as percutaneous liver biopsy. Percutaneous liver biopsy revealed the findings consistent with cholestasis, minimal portal and lobular inflammatory infiltration, and minimal steatosis (Figure 1). Inguinal lymph node biopsy findings were consistent with classical Hodgkin's disease with mixed cellularity (CD30, CD15, and fascin positive) (Figure 2). Upon the diagnosis of stage II Hodgkin's disease, patient was referred to the oncology department for chemotherapy. Two weeks after his referral to oncology department, the patient died due to hepatic failure.

## 3. Discussion

The diagnosis of Hodgkin's disease in our patient presenting with the complaint of jaundice required extensive work-up including surgical procedures until the exclusion of bile duct obstruction or extrahepatic causes of cholestasis and identification of inguinal lymph node biopsy findings consistent with classical HL with mixed cellularity and liver biopsy findings consistent with intrahepatic cholestasis.

In our case, jaundice developed in the absence of any medication or herbal remedy and all alternative diagnoses have been ruled out. Besides, pathological findings were compatible with the cases previously defined in literature [7] and in agreement with the statement that the histopathologic findings in idiopathic cholestasis are nonspecific and most commonly show canalicular stasis with mixed inflammatory infiltrates [8]. Therefore we can suggest that there is a direct relationship between the HL and idiopathic cholestasis in our patient.

Given that idiopathic intrahepatic cholestasis as a presenting symptom of HL has been rarely documented in the medical literature [1, 5–7], our findings emphasize the consideration of HL-related idiopathic intrahepatic cholestasis as a diagnosis of exclusion in case of the evident significant hepatic dysfunction along with distinct histological findings, while lacking any evidence on liver involvement of HL and other known causes of liver disease [7].

When cholestasis-related clinical signs and symptoms are observed in patients with HL, our findings suggest considering possibility of either VBDS or idiopathic cholestasis in the diagnostic procedures.

The exact pathogenesis of paraneoplastic syndrome-related cholestasis has not yet been clarified, while the emission of a cholestatic cytokine or elaboration of hormones such as androgens or 17-alkylated estrogens from the tumor cells has been proposed amongst the possible mechanisms [9]. Nonetheless, given the better clinical course associated with idiopathic form, timely diagnosis of HL-related intrahepatic cholestasis with proper discrimination of VBDS and idiopathic form seems to be of utmost importance.

Notably, diagnosis of both VBDS and idiopathic intrahepatic cholestasis seems to be crucial given that these two paraneoplastic phenomena are distinct not only in terms of histological characteristics but also in their clinical course with better prognosis in case of idiopathic intrahepatic cholestasis [6].

In this regard, given that patients with idiopathic cholestasis generally have a better prognosis when presenting with stage I/II HL and when treated with radiation [6], considering diagnostic work-up carried out from initial admission to final diagnosis of stage II HL, response of cholestasis to the treatment cannot be assessed due to short survey of the patient. In this case detection of inguinal lymphadenopathy only after the surgical operation and delay in liver biopsy results in hepatic failure and death of the patient. PET-CT scan could reveal pathologic lymph nodes before physical examination and unfortunately financial status of patient was insufficient. Our findings signify the value of physical examination as well as early liver biopsy in the differential diagnosis of the jaundice.

In conclusion, given that jaundice is a commonly faced sign indicative of a large list of diseases in the clinical practice, our findings suggest the consideration of HL-related idiopathic intrahepatic cholestasis as a diagnosis of exclusion in patients presenting with jaundice of indefinite origin and signify the value of early liver biopsy in the differential diagnosis of the jaundice.

## Figures and Tables

**Table 1:** Blood biochemistry findings for the parameters related to liver function.

	Jan 2012	Feb 2012	Mar 2012
Aspartate aminotransferase (U/L)	242	130	89
Alanine aminotransferase (U/L)	228	112	42
Gamma-glutamyltransferase (U/L)	176	98	64
Alkaline phosphatase (U/L)	402	398	407
Lactate dehydrogenase (U/L)	704	608	419
Total bilirubin (mg/dL)	6.38	12.9	17.29
Direct bilirubin (mg/dL)	0.39	8.9	14.01
Albumin (g/dL)	4	3.6	3.4
